# Malnutrition and Its Influence on Gut sIgA–Microbiota Dynamics

**DOI:** 10.3390/biomedicines13010179

**Published:** 2025-01-13

**Authors:** Monica Profir, Robert Mihai Enache, Oana Alexandra Roşu, Luciana Alexandra Pavelescu, Sanda Maria Creţoiu, Bogdan Severus Gaspar

**Affiliations:** 1 Department of Morphological Sciences, Cell and Molecular Biology and Histology, Carol Davila University of Medicine and Pharmacy, 050474 Bucharest, Romania; monica.profir@rez.umfcd.ro (M.P.); oana-alexandra.rosu@rez.umfcd.ro (O.A.R.); luciana.pavelescu@umfcd.ro (L.A.P.); 2 Department of Oncology, Elias University Emergency Hospital, 011461 Bucharest, Romania; 3 Department of Radiology and Medical Imaging, Fundeni Clinical Institute, 022328 Bucharest, Romania; robert-mihai.enache@rez.umfcd.ro; 4 Department of Surgery, Carol Davila University of Medicine and Pharmacy, 050474 Bucharest, Romania; bogdan.gaspar@umfcd.ro; 5 Surgery Clinic, Bucharest Emergency Clinical Hospital, 014461 Bucharest, Romania

**Keywords:** malnutrition, gut microbiota, obesity, overweight, underweight, secretory IgA

## Abstract

In the current era, malnutrition is seen as both undernutrition and overweight and obesity; both conditions are caused by nutrient deficiency or excess and improper use or imbalance in the intake of macro and micronutrients. Recent evidence suggests that malnutrition alters the intestinal microbiota, known as dysbiosis. Secretory immunoglobulin A (sIgA) plays an important role in maintaining and increasing beneficial intestinal microbiota populations and protecting against pathogenic species. Depletion of beneficial bacterial populations throughout life is also conditioned by malnutrition. This review aims to synthesize the evidence that establishes an interrelationship between diet, malnutrition, changes in the intestinal flora, and sIgA levels. Targeted nutritional therapies combined with prebiotic, probiotic, and postbiotic administration can restore the immune response in the intestine and the host’s homeostasis.

## 1. Introduction

**Figure 1:**
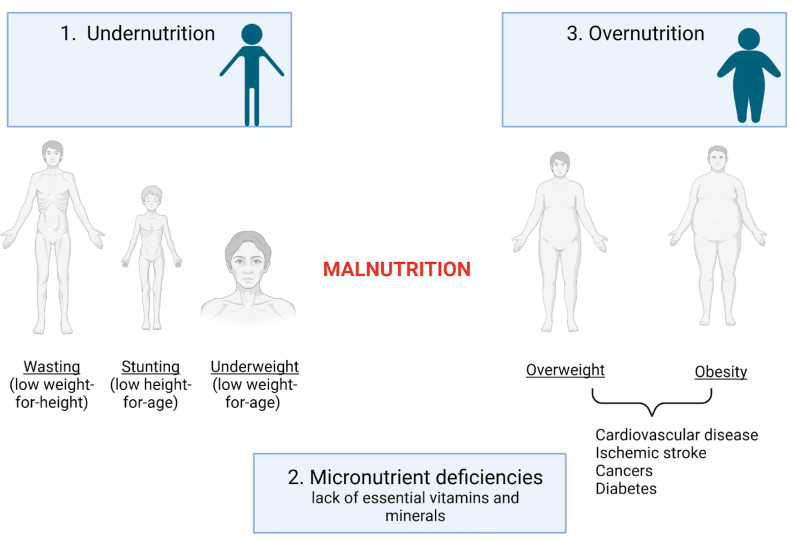
Categories of malnutrition. Undernutrition: includes wasting (low weight-for-height), stunting (low height-for-age), and underweight (low weight-for-age). Micronutrient deficiencies: refers to a lack of essential vitamins and minerals. Overnutrition: encompasses overweight, obesity, and diet-related noncommunicable diseases. Created using BioRender.com (accessed on 22 October 2024).

According to the World Health Organization (WHO), malnutrition encompasses deficiencies, excesses, or imbalances in a person’s intake of energy and nutrients [1]. It is classified into three main categories: undernutrition (including wasting, stunting, and underweight), micronutrient-related malnutrition (involving deficiencies or excesses of essential vitamins and minerals), and conditions like overweight, obesity, and diet-related noncommunicable diseases, such as cardiovascular disease, ischemic stroke, certain cancers, and diabetes [1]. They also emphasized the issue that 60 million adults and 150 million children are malnourished, while over two billion adults and children are classified as overweight or obese [2]. In Figure 1, we depict the main categories of malnutrition as defined by the WHO.

Undernutrition is defined as insufficient energy and nutrients necessary to maintain stable health, increasing the risk of illness and death in children. It is classified into the following categories [1]:

- Wasting: characterized by low weight-for-height, indicating that a child’s weight is significantly below the standard compared to a reference population. Also known as global undernutrition or global acute malnutrition, wasting is often caused by insufficient food or infectious diseases causing diarrhea, which, in moderate-to-severe cases, increases the risk of mortality in children—though it is treatable. Severe wasting affects approximately 13.7 million children under five globally, substantially increasing the risk of mortality if not promptly addressed [3];
- Stunting: defined as low height-for-age where a child’s stature is markedly shorter than that of peers in a reference population. Stunting results from chronic or repeated undernutrition in families facing poor socioeconomic conditions, inadequate maternal health and nutrition, recurrent illness, or improper infant feeding, which limits a child’s physical and cognitive potential. Despite a steady decline since 2000, stunting remains a significant concern, with 22.3% of all children under five affected in 2022 [3];
- Underweight: identified as low weight-for-age, indicating that a child’s weight is substantially below the standard for their age in a reference population. Being underweight can result from any single form of undernutrition or a combination of wasting and stunting. Undernutrition contributes to nearly half of all deaths in children under five, as it increases susceptibility to common infections, delays recovery, and exacerbates the severity of disease [4].

Micronutrient deficiencies, commonly called “hidden hunger”, are significant global health concerns, particularly among children and pregnant women in low- and middle-income regions. Deficiencies in essential nutrients such as iodine, vitamin A, and iron can severely impede physiological processes critical for growth, development, and overall health. Recent studies indicate that approximately 2 billion people globally suffer from micronutrient deficiencies, exacerbated by food insecurity and climate change impacts on agriculture. Integrated approaches, including food fortification, supplementation programs, and dietary diversification, are vital to addressing these deficiencies and improving population health [3].

- Iodine deficiency is the leading cause of preventable intellectual disability worldwide [3];
- Vitamin A deficiency compromises immune function, increasing the risk of severe infections in children [3];
- Iron deficiency, the most prevalent micronutrient deficiency, leads to anemia, which affects cognitive and physical development and reduces productivity in adults [3].

Overweight and obesity are conditions characterized by excess body weight relative to height, resulting from an imbalance between calorie intake and energy expenditure. These conditions are classified based on the body mass index (BMI): a BMI of 25 or higher indicates overweight, while a BMI of 30 or more signifies obesity. In children, BMI thresholds vary with age and sex, requiring age-specific percentile charts for accurate classification. The rise in overweight and obesity rates is primarily driven by increased consumption of energy-dense foods and beverages high in sugars and fats, coupled with sedentary lifestyles. Globally, childhood obesity has reached alarming levels, with over 39 million children under five classified as overweight or obese in 2022. These conditions are associated with a heightened risk of diet-related noncommunicable diseases, including type 2 diabetes, cardiovascular diseases, and certain cancers. The economic burden of obesity is also substantial, with healthcare costs and lost productivity imposing significant challenges on global health systems. Preventive strategies, such as promoting balanced diets, increasing physical activity, and implementing policies to regulate unhealthy food marketing, are critical to reversing this trend [5].

Malnutrition can stem from various environmental, economic, and medical conditions. The most common causes include the following:

1. Limited access to sufficient and affordable food in both developing and developed countries [2]. Research shows that poverty is linked to poor nutrition in preschool children, while in adults and the elderly, food insecurity and poverty contribute to malnutrition [6]. Studies have also connected food insecurity to a higher risk of diarrhea, respiratory infections, and parasitic diseases in children, leading to stunting and underweight cases in proportion to its severity [7]. Additionally, food insecurity is independently associated with overweight conditions [8];
2. Digestive disorders, such as Crohn’s disease, celiac disease, nonsteroidal-induced enteropathy, and other conditions associated with dysbiosis, are common causes of malnutrition [2]. Research has highlighted that undernutrition, especially in patients with inflammatory bowel disease (IBD), can be a marker for poor prognosis [9,10];
3. Excessive alcohol consumption can result in malnutrition, particularly in developed countries [1]. Chronic alcohol intake leads to liver disease, pancreatitis, and secondary malnutrition due to protein and nutrient deficiencies, with its severity linked to the degree of liver impairment and the risk of morbidity and mortality [11,12];
4. Mental health disorders, especially depression, have been linked to an increased risk of malnutrition in older adults, in addition to vegetative symptoms [1,13];
5. Physical disabilities also contribute to malnutrition [1]. Studies indicate that geriatric males, particularly those with frailty marked by lower BMI, decreased muscle mass, reduced strength, and sarcopenia, are at higher risk [14]. There is a strong interconnection between malnutrition and physical disability [15];
6. Increased nutritional needs during pregnancy and lactation are another contributing factor. Malnutrition is particularly prevalent among teenage pregnancies and during the breastfeeding period [1,16,17].

## 2. Gut Microbiota and Factors Influencing Its Diversity

**Figure 2:**
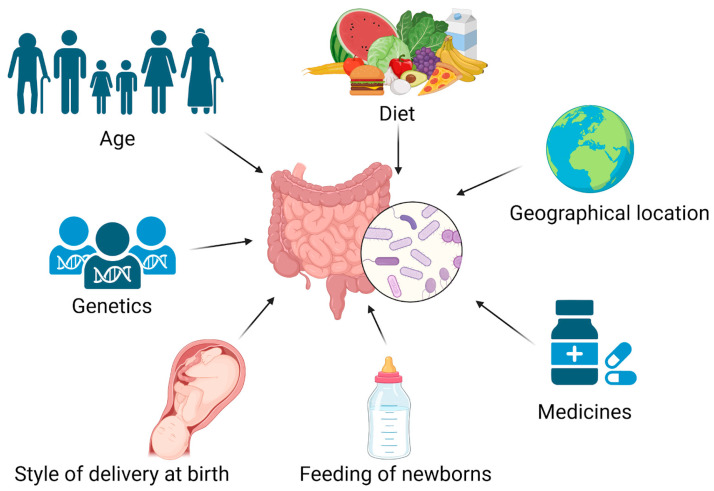
The most important factors that influence the gut microbiota composition in humans. Created using BioRender.com (accessed on 30 November 2024).

The human microbiota exhibits far more diversity than human cells, complicating efforts to comprehend how microorganisms and their metabolites influence health and contribute to disease development [18]. It is difficult to fathom how bacteria and their metabolites impact health and contribute to the development of illness since the human microbiota displays a greater degree of variety than human cells do [19]. The colonization, growth, composition, and diversity of the microbiota in the gut are all influenced by many different circumstances. The most important factors that determine the colonization and variety of microorganisms are illustrated in Figure 2 and include age, genetics, the style of delivery at birth, the ways of feeding newborns, medicines (such as antibiotics), geographical location, and diet [20,21,22,23,24,25,26,27,28,29,30,31].

In the time leading up to delivery, the fetal intestine is inhabited by the mother’s microbiota. It is during this crucial period of one thousand days that the microbiota in the gut grows and achieves a composition that is comparable to that of an adult [32]. The estimated quantity of microbial cells in the human body is almost equivalent to that of human cells [18]. The mother’s microbiota colonizes the fetal gut before the infant is born. A child is exposed to a wide range of microbiota from birth, and milk-associated bacteria are passed on to the newborn who is breastfeeding. Taking everything into account, maternal food has the capacity to govern the makeup of the mother’s microbiota, as well as that of the newborn and child being fed [33].

Micronutrient deficiencies during the first thousand days may affect the maturation of the microbiota and its interactions with the host, which in turn affects development throughout adolescence and adulthood. Epigenetic mechanisms may significantly impact the relationships between bacteria and hosts. Inadequate consumption of micronutrients (vitamins A and D, iron, and zinc) and one-carbon sources (folate and vitamin B12) in the diet, which often happens in both low- and high-income nations, may impact microbiota maturation during the 1000-day window.

The gut bacterial community makeup of babies delivered by cesarean section differs from that of infants born via vaginal birth [34,35]. At birth, infants who are delivered vaginally are exposed to the bacteria that are present in the mother’s body. This has the effect of influencing the bacteria that are present in the infant’s stomach and stimulating cells of the immune system [36].

Infant feeding plays a crucial role in the formation of the gut bacterial population since maternal milk is not sterile [37]. Human breast milk is recognized as a source of commensal bacteria, as well as possibly probiotic bacteria, which have an impact on the development of the gut microbiota of premature infants [38]. Among the variables that have the greatest impact on the formation and modification of the human gut microbiota are dietary factors, which include both micro- and macronutrients [39]. Malnutrition causes a persistent disturbance in the development of the intestinal microbiota in children, which leads to continued and often severe malnutrition. This occurs even though nutritional therapy in the form of ready-to-use food that contains both macro- and micronutrients is being administered [40]. There is evidence from research conducted on both humans and animals suggesting a higher ratio of *Firmicutes* to *Bacteroidetes* (F/B ratio) is linked to phenotypes of obesity and leanness and that this ratio may also influence energy balance [41,42]. Research is required to investigate the interactions of food, dietary components, and microbiota in hidden hunger and undernutrition. A link exists between the host’s genetic composition and the quantity of certain bacteria in the gut microbiota. This association may affect the host’s metabolism, thus influencing health [43]. Compared to people who are unrelated to one another, members of the same family have a greater degree of similarity in their microbial communities. Additionally, the gut microbiota of monozygotic twins is more similar than that of dizygotic twins [43].

There is a correlation between the microbiota in the gut and the presence of viral and bacterial disorders, and infections can influence the microbiota [44,45,46]. A growing corpus of research indicates that several non-antibiotic drugs influence the gastrointestinal microbiota [47,48]. Metformin led to a larger abundance of the mucin-degrading bacteria *Akkermansia* in obese mice than in their obese counterparts who were not given metformin, according to the findings of research in which obesity was created in mice by giving them a diet that was rich in fat [49]. Metaformin’s influence on the microbiota in the gut was proven by a recent study conducted on humans [50].

## 3. Gut Microbiota in Undernutrition

**Figure 3:**
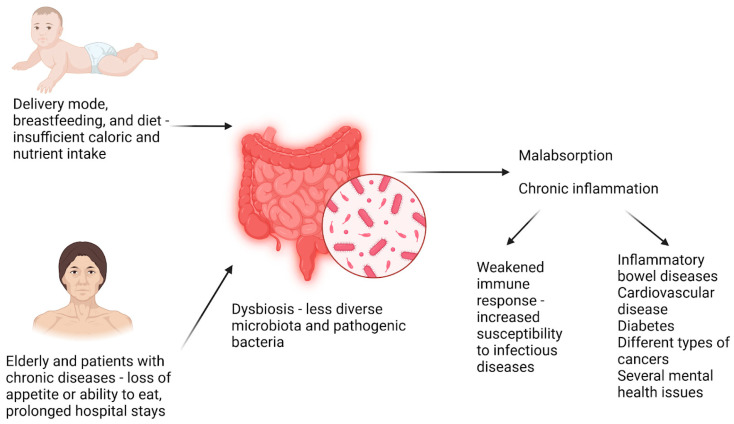
Undernutrition predominantly affects two vulnerable groups: newborns and the elderly, as well as patients with chronic diseases. This condition often results in dysbiosis, characterized by malabsorption and chronic inflammation, significantly increasing the risk of immune dysfunction and other health complications. Created using BioRender.com (accessed on 30 November 2024).

Undernutrition, a form of malnutrition, is a threatening condition in children and adults. Undernutrition is more frequently observed in children from developing countries, and it accounts for approximately half of the deaths among children under 5 years old. In 2022, the WHO reported that, globally, 390 million adults were underweight, 149 million children were stunted (too short for their age), and 45 million children were wasted (too thin for their height) [1]. Undernutrition has four forms: wasting, stunting, being underweight, and deficiencies in vitamins and minerals. All of these are known to have a negative impact on general health and predispose children to developing diseases [51]. Figure 3 illustrates the effects of undernutrition on the most vulnerable age groups.

Undernutrition, especially in early life, profoundly impacts the development and composition of the gut microbiota, with lasting consequences for health and immunity. This state, characterized by insufficient caloric and nutrient intake, leads to stunted growth, impaired cognitive development, and a weakened immune response, making individuals more susceptible to infectious diseases. Evidence suggests that the gut microbiota of undernourished individuals, especially children, is significantly less diverse and often harbors pathogenic bacteria that can further exacerbate nutritional deficiencies and immune dysfunctions [52]. In adults, undernutrition is more common in the elderly and patients with chronic diseases due to increased caloric absorption, loss of appetite or ability to eat, and even prolonged hospital stays [53].

Dysbiosis, the alteration of the gut microbial composition, has been associated with several diseases, including IBD, cardiovascular disease, diabetes, different types of cancers, and several mental health issues [54,55,56,57,58]. Various forms of malnutrition have also been linked to the alteration of the gut microbiota, representing both a cause and an effect of malnutrition [59]. The gut microbiota plays a key role in metabolism, absorption of nutrients and vitamins, and general health. Thus, a healthy gut microbiota is crucial for the optimal absorption of nutrients. Alterations to the microbial composition of the intestinal microbiota can modify the way food is processed and the production of vitamins by the gut microbiota, leading to malabsorption even with proper food intake. In addition, dysbiosis is frequently associated with chronic inflammation and is linked to disease-related malnutrition, anorexia, and muscle catabolism [60]. Pathogenic bacteria can also have a negative impact on nutrient absorption by causing intestinal epithelial damage and severe forms of diarrhea [61].

As previously stated, malnutrition can also be a cause of dysbiosis. Hashimoto et al. demonstrated that a protein-free diet can lead to tryptophan deficiency, causing a drop in vitamin B3 levels, which reduces the production of antimicrobial peptides in the ileal epithelium. This leads to alterations in the microbial community in the colon, which causes epithelial damage, triggers a harmful inflammatory response, and results in diarrhea. By transplanting this altered microbiota into germ-free mice, researchers confirmed that the intensified epithelial colitis was directly related to this modified microbial community [62].

The establishment of a healthy gut microbiota begins at birth and is influenced by factors such as delivery mode, breastfeeding, and diet. By the age of 2 to 3 years, a child’s gut microbiota typically matures, resembling that of an adult in diversity and composition [63]. However, in malnourished children, this natural progression is often disrupted. Studies on severely malnourished children have found that their gut microbiota resembles that of younger, undeveloped microbiomes, lacking essential bacteria associated with carbohydrate and protein metabolism, such as *Bifidobacterium* and *Bacteroides* [64]. This microbial immaturity compromises nutrient absorption and energy utilization, further deepening the nutritional deficit. In their review, Zoghi et al. examined the gut microbiota and dysbiosis in children with undernutrition and overnutrition. They concluded that an imbalanced microbial environment contributes to malnutrition and highlighted that restoring gut microbial homeostasis through innovative therapeutic approaches—such as probiotics, prebiotics, synbiotics, postbiotics, fecal microbiota transplantation (FMT), and bioengineering methods—can mitigate the adverse effects of childhood malnutrition [65]. Similarly, Iddrisu et al. emphasized the role of diarrhea in the progression of malnutrition among children. They explained that diarrhea impairs nutrient absorption and reduces energy availability, exacerbating malnutrition. Additionally, they noted that children with severe malnutrition exhibit significant alterations in their gut microbiota, including an increase in *Proteobacteria*, a decrease in *Bacteroides*, and reduced microbial diversity. Their findings underscore the critical role of diet in shaping the gut microbiota of malnourished children, particularly given the rapid changes in microbiota composition during early childhood [66].

Studies often find more inflammation-associated bacteria in children with severe malnutrition, like *Enterobacteriaceae*, are linked to an increased “leaky gut” phenomenon. For example, a study in Malawi found that malnourished children had more potentially harmful bacteria, including *Shigella* and *Campylobacter*, which weaken the gut’s barrier function [67]. When the gut barrier is compromised, it becomes easier for bacteria and their toxins to pass through the gut lining into the bloodstream, leading to more inflammation and making it harder for the body to absorb nutrients. It creates a cycle where poor nutrition harms the gut microbiota, and unhealthy microbiota worsens nutritional deficiencies. The immune system is negatively affected when the gut microbiota is compromised, and malnourished individuals are more prone to gastrointestinal infections. For example, children who have fewer beneficial *Lactobacillus* and *Bifidobacterium* species are more prone to gastrointestinal infections, a serious issue in malnourished populations [67]. Also, a review by Vemuri et al. highlighted that gut microbiota composition undergoes significant changes with aging. These changes are primarily characterized by an imbalance between anaerobic *Firmicutes* and *Bacteroidetes*, alongside an increase in various facultative organisms, collectively contributing to an impaired immune system in older adults. The weakened immune response allows for the proliferation of pathogens in the gut, resulting in low-grade chronic inflammation and a heightened risk of various diseases. The authors also noted that frequent antibiotic use among the elderly, often due to infections, exacerbates microbiota imbalances. They emphasized that these age-related alterations in gut microbiota can contribute to malnutrition but are potentially reversible. Strategies such as careful management of antibiotic use, supplementation with prebiotics and probiotics, and, in certain cases, FMT, can help restore microbial balance and mitigate the associated risks [68].

Different forms of undernutrition are associated with environmental enteric dysfunction (EED), an intestinal condition marked by shortened villi, inflammation, decreased barrier function, and other enteropathy characteristics. EED is common in low-income areas where sanitation is limited and exposure to pathogens is high [69]. EED leads to chronic gut inflammation and increased permeability, making it difficult to absorb nutrients. Studies suggest that EED-related dysbiosis is characterized by a lack of certain beneficial bacteria that are important for nutrient uptake and gut health, such as *Bifidobacterium* and *Lactobacillus* species [70]. In areas with high EED prevalence, children often have gut microbiota profiles dominated by inflammation-associated bacteria, exacerbating malabsorption issues [71]. The gut microbiota of children with EED differs significantly from those of healthy, well-nourished children, but no studies have aimed to characterize the intestinal microbiota in patients with EED. This relationship highlights the importance of combining efforts to address environmental health and gut microbiota restoration in undernutrition strategies. The key findings of the most important studies regarding malnourished pediatric populations are included in Table 1.

## 4. Gut Microbiota in Obesity

Obesity is a form of malnutrition, a complex health condition influenced by several factors, including genetics, lifestyle, and environmental elements. In 2022, WHO reported that 2.5 billion adults were overweight, and 890 million adults and 37 million children under 5 years were living with obesity [1]. Obesity is associated with increased morbidity and mortality rates, along with higher healthcare costs, making it one of the greatest global health challenges [1].

In recent years, scientists have focused on understanding the role of gut microbiota in influencing body weight, metabolic health, and obesity-associated inflammation. The gut microbiota of people with obesity is often distinct from that of lean individuals, characterized by shifts in bacterial diversity and composition [74]. Studies consistently show that obesity is associated with increased relative abundance of the bacterial phylum Firmicutes and reduced *Bacteroidetes* abundance [41,75]. For example, in a pioneering study by Ley et al., researchers observed that the Firmicutes-to-Bacteroidetes ratio was significantly higher in obese individuals than their lean counterparts, suggesting a link between this altered ratio and obesity [41]. This change in microbial composition is believed to enhance the capacity for energy harvest from the diet, contributing to excess calorie absorption and fat storage [75]. In addition to changes in these major bacterial phyla, obesity is associated with a decrease in the abundance of beneficial bacterial genera such as *Bifidobacterium* and *Akkermansia* [76,77]. *Akkermansia muciniphila*, in particular, is known for its role in maintaining gut barrier integrity and regulating inflammation. Lower levels of *Akkermansia muciniphila* are associated with increased gut permeability, which can lead to the development of a “leaky gut.” This increased permeability allows bacterial products, such as lipopolysaccharides (LPSs), to enter the bloodstream, triggering systemic inflammation—a hallmark of metabolic diseases, including obesity and type 2 diabetes [78]. The administration of *Akkermansia* in rodents has led to reduced insulin resistance, decreased steatosis, and excess weight [79].

One of the key mechanisms by which the gut microbiota influences body weight is through its role in breaking down complex carbohydrates and extracting energy from food. Certain bacteria in the gut, particularly those from the *Firmicutes* phylum, possess enzymes that can break down otherwise indigestible polysaccharides into short-chain fatty acids (SCFAs) like butyrate, propionate, and acetate, which can serve as an energy source for the body and can also stimulate fat storage in adipose tissues [80]. Studies have shown that individuals with obesity have higher levels of SCFA-producing bacteria, which likely contributes to their increased caloric intake even without consuming additional food [75].

Moreover, certain gut bacteria in obese individuals can increase energy absorption efficiency, leading to greater calorie intake from the same amount of food. This phenomenon was demonstrated in germ-free mice transplanted with microbiota from obese and lean humans. The mice that received microbiota from obese humans gained significantly more weight and fat than those transplanted with microbiota from lean humans despite consuming the same diet [81]. This experiment highlighted the gut microbiota’s capacity to impact energy balance and fat storage independently of diet. Table 2 synthesizes the most important findings of studies on human and animal populations or models.

Chronic, low-grade inflammation is a characteristic of obesity and is thought to be partly driven by the gut microbiota. In particular, the increase in gut permeability in obese individuals allows microbial byproducts, such as LPSs from Gram-negative bacteria, to translocate from the gut into the bloodstream. This process, known as “metabolic endotoxemia”, can trigger inflammatory responses throughout the body, promoting insulin resistance and fat accumulation. Cani et al. demonstrated that mice fed a high-fat diet experienced a significant increase in circulating LPS levels, which correlated with weight gain and inflammation. This inflammatory response exacerbates the metabolic dysfunctions associated with obesity, making it more difficult for individuals to lose weight [78]. Another inflammatory pathway involves the production of bile acids, which are regulated by gut bacteria. The bile acids aid in fat digestion and serve as signaling molecules that interact with metabolic pathways. Certain gut bacteria can alter bile acid profiles, promoting inflammation and impairing glucose metabolism, thereby contributing to obesity-related metabolic disturbances [82].

To facilitate the understanding of how undernutrition and overnutrition affect sIgA levels and gut microbiota, the key differences and similarities are outlined in Table 3. This table shows how these two forms of malnutrition influence gut health, from changes in microbial diversity to gut barrier integrity.

## 5. The Significance of Fecal sIgA Related to Microbiota Diversity

sIgA plays an essential role in maintaining gut health by serving as a key immune component in the mucosal lining of the gastrointestinal tract. As the most abundant antibody in mucosal areas, sIgA interacts directly with gut microbes, binding to potentially harmful pathogens and preventing them from attaching to the gut lining [83]. Additionally, sIgA influences the composition and diversity of the gut microbiota by shaping which microbial species can thrive in the gut environment [84]. Recent studies indicate that sIgA can support microbial diversity, encourage beneficial species, and reduce pathogenic organisms—functions that are critical to maintaining a balanced and healthy microbiota [84].

sIgA is produced by immune cells located in the gut’s mucosal tissue and is continuously secreted into the intestinal lumen, where it interacts with the microbiota [85]. The gut microbiota is a dynamic environment with a wide range of bacterial species, and sIgA plays a unique role in maintaining balance among them. By coating certain bacterial species, sIgA can prevent pathogenic microbes from colonizing the gut wall while promoting the growth of commensal and beneficial bacteria [86]. This selective binding creates a healthier microbial environment that promotes diversity, protects against harmful pathogens, and supports the integrity of the intestinal lining [87].

The ability of sIgA to “neutralize” potentially harmful microbes without triggering inflammation is one of its distinguishing characteristics. Unlike other immune responses, which can lead to inflammation and damage to host tissues, sIgA’s activity is “non-inflammatory” [88]. It binds to bacteria in the gut lumen, preventing their adhesion to the epithelial layer without stimulating an aggressive immune reaction [89]. This “immune exclusion” mechanism allows sIgA to promote a non-inflammatory tolerance in the gut, which is crucial for a diverse, healthy microbiota.

sIgA plays a major role in maintaining immune tolerance in the gut, which is essential given that the gut must coexist with many non-pathogenic microbes [87]. This tolerance is partly achieved through sIgA’s “immune training” of the gut microbiota. In early life, for example, sIgA coating helps the immune system learn which bacterial species are commensal and non-threatening, fostering a microbiota that remains diverse and stable over time [90]. Research indicates that lower levels of sIgA can lead to an imbalance in the microbiota, increasing susceptibility to infections, inflammation, and even autoimmune conditions [91].

Additionally, the diversity of sIgA-bound bacteria has been linked to broader diversity in the gut microbiota. This relationship appears to be reciprocal, with increased microbiota diversity promoting a stronger sIgA response, which in turn further supports microbiota stability [92]. Studies have shown that people with reduced sIgA levels tend to have a less diverse microbiota, which can leave them more vulnerable to dysbiosis—a condition where harmful bacteria outgrow beneficial ones, leading to health complications ranging from inflammation to metabolic issues [93].

**Figure 4:**
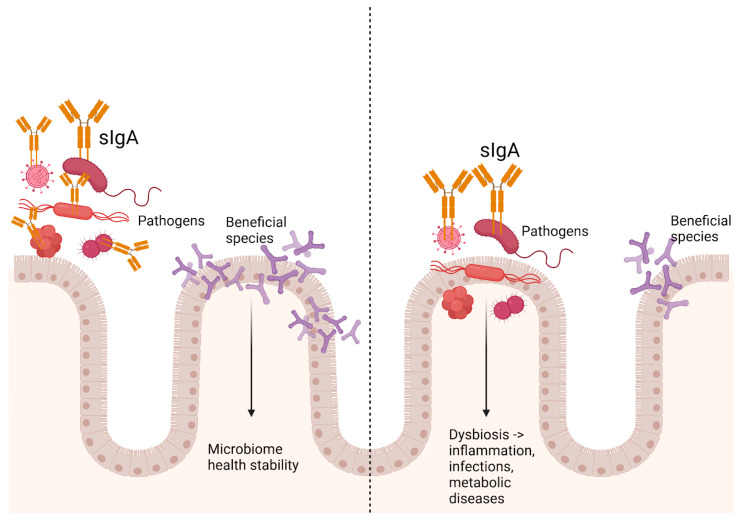
The unique role of sIgA in maintaining gut homeostasis. It helps regulate microbial communities and fortifies the mucosal barrier by directly interacting with gut microbes. sIgA binds to potentially harmful pathogens, preventing their adhesion to the gut lining while promoting the presence of beneficial microbial species. Dysregulation of sIgA can disrupt this balance, leading to microbial imbalances where harmful bacteria dominate, contributing to or exacerbating disease states. Created using BioRender.com (accessed on 30 November 2024).

Interestingly, fecal sIgA levels can fluctuate depending on a person’s nutritional status, diet quality, and lifestyle factors. Undernutrition and nutrient deficiencies are known to weaken immune functions overall, including the production of sIgA [73]. Studies have found that children with undernutrition often show lower levels of fecal sIgA, which correlates with reduced microbial diversity in their gut [94]. These children are often more susceptible to infections and inflammation, possibly due to a weaker gut barrier and a less diverse microbiota. Undernutrition, therefore, can contribute to a vicious cycle where low sIgA levels decrease microbiota diversity, further impairing immune function and leaving individuals vulnerable to additional infections [95]. Figure 4 illustrates the role of sIgA in modulating gut microbiota under both healthy and pathological conditions.

**Figure 5:**
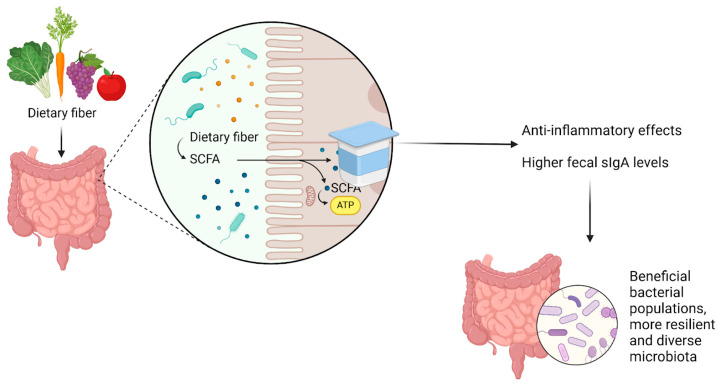
Dietary interventions and nutritional supplements enhance fecal sIgA levels, promoting a diverse and healthy gut microbiota. Diets rich in fiber and fermented foods like yogurt and kimchi contribute to this effect. One of the mechanisms involved is the production of SCFAs, which possess anti-inflammatory properties and help maintain gut homeostasis. Created using BioRender.com (accessed on 30 November 2024).

In contrast, certain dietary interventions and nutritional supplements can increase fecal sIgA levels, promoting microbiota diversity as illustrated in Figure 5 [96,97]. Diets high in fiber, for instance, have been shown to encourage the growth of bacteria that produce SCFAs, which have anti-inflammatory effects and support gut health [98]. SCFA production has also been linked to higher fecal sIgA levels [99]. Similarly, the consumption of fermented foods rich in probiotics, such as yogurt and kimchi, can help boost beneficial bacterial populations and sIgA levels, fostering a more resilient and diverse microbiota [100].

The interaction between sIgA and gut microbiota diversity has critical implications for several health conditions. Lower fecal sIgA levels, for example, are associated with IBD and irritable bowel syndrome (IBS), both characterized by microbial imbalances and increased gut permeability [93]. Individuals with these conditions often exhibit altered sIgA responses, possibly contributing to ongoing inflammation and dysbiosis [101]. Clinical studies have shown that patients with IBD often have reduced levels of sIgA-bound bacteria, which correlates with lower microbial diversity and higher disease activity [102]. sIgA and microbiota diversity are also relevant in metabolic diseases like obesity and type 2 diabetes. Lower microbiota diversity and compromised gut barrier function have been observed in individuals with obesity, who also tend to have reduced levels of sIgA. This deficiency in sIgA can lead to increased gut permeability and systemic inflammation, further exacerbating metabolic issues [103]. Research suggests that restoring sIgA levels through dietary changes or microbiota-targeted therapies might support microbiota diversity and potentially improve metabolic health. Kau et al. investigated the interplay between childhood undernutrition, gut microbiota, and gut mucosal immune function, focusing on sIgA-targeted bacterial taxa. Using fecal samples from children during their first two years of life, they employed a technique called BugFACS to analyze sIgA-bound microbes. Their findings revealed that sIgA responses to specific bacteria, including *Enterobacteriaceae*, were associated with undernutrition [104]. Moreover, transplanting sIgA-bound bacteria from undernourished children into germ-free mice induced diet-dependent enteropathy and malnutrition, which could be mitigated by providing a nutrient-sufficient diet or introducing two sIgA-targeted bacterial species from the microbiota of a healthy donor. The study concluded that sIgA-targeted bacterial taxa have significant etiologic, diagnostic, and therapeutic implications for addressing childhood undernutrition [104].

Malnutrition significantly alters the immune and metabolic signaling networks within the gut, leading to disruptions in both microbial diversity and sIgA production. Several mechanisms and pathways including the mammalian target of rapamycin (mTOR), Toll-Like Receptors (TLRs), provide insights into how these effects are mediated.

The mTOR pathway is crucial for maintaining immune cell function, including plasma cells that produce sIgA. Nutritional deficiencies, especially protein and essential amino acids, downregulate the mTOR pathway, impairing the differentiation and function of B cells and plasma cells [105]. The mTOR pathway is highly sensitive to amino acid availability, and protein deficiencies lead to reduced immune function [106]. In a state of malnutrition, reduced amino acid supply compromises mTOR’s ability to support the metabolic demands of immune cells, including B cells and plasma cells, which are crucial for producing secretory immunoglobulin A (sIgA) [107]. However, direct evidence specifically linking reduced mTOR activation to decreased levels of secretory sIgA is limited, and further evidence is needed to elucidate this specific connection.

TLRs play a crucial role in recognizing microbial patterns and initiating immune responses. Protein and amino acid deficiencies can downregulate the expression and functionality of TLRs in the gut. This impairment disrupts the MyD88/NF-κB signaling pathway, essential for cytokine production and mucosal immunity [107]. A weakened TLR response compromises the ability to regulate gut microbiota composition effectively, resulting in dysbiosis [108,109]. Furthermore, malnutrition reduces the immune system’s capacity to produce secretory immunoglobulin A (sIgA), a key antibody responsible for coating gut microbes and preventing the overgrowth of pathogenic bacteria [72].

The nuclear factor kappa-light-chain-enhancer of activated B cells (NF-κB) is essential for initiating immune responses and promoting IgA class switching in B cells. Nutrient deficiencies, especially in vitamins A and D, can impair NF-κB activation [103]. Vitamin A deficiency reduces the production of retinoic acid, which is essential for IgA synthesis [110]. Dysregulation of NF-κB signaling due to nutritional deficiencies decreases sIgA production, leading to a less effective gut barrier and reduced microbiota diversity [73].

## 6. The Role of Diet, Adequate Micronutrients, and Bacterial Supplementation

The World Health Organization (WHO) provides substantial evidence supporting the safety and health benefits of consuming specific probiotic strains. Foods rich in probiotics, such as yogurt, cheese, and milk, or supplements like lozenges and chewing gum, have demonstrated potential advantages for human health [111].

### 6.1. Gut Microbiota Restitution

#### Probiotics, Prebiotics, and Synbiotics

Probiotics are live microorganisms that provide health benefits when consumed in the right amounts. The WHO and the Food and Agriculture Organization of the United Nations officially defined them in 2001, recognizing their important role in supporting health [104]. Research has repeatedly confirmed that probiotics are safe and effective, helping to prevent and treat a wide range of health issues like acute diarrhea, inflammatory disorders, cardiovascular diseases, urogenital infections, dental issues, and even certain cancers [112,113]. Probiotics work by encouraging the growth of beneficial bacteria while suppressing pathogenic species [112]. They boost the immune system by increasing the production of antimicrobial compounds and competing with pathogens for space in the gut [114,115,116]. Research has shown that certain probiotic strains, such as *Lactobacillus* and *Bifidobacterium*, can temporarily colonize the gut, providing lasting benefits even after they are no longer consumed [115]. Importantly, probiotics have been linked to increased secretory immunoglobulin A (sIgA) secretion, which plays a key role in gut mucosal immunity by neutralizing pathogens and toxins [87]. A clinical trial involving 45 obese patients investigated the effects of a low-carbohydrate, reduced-energy diet alone versus the same diet supplemented with either prebiotics or probiotics (a daily tablet containing *Bifidobacterium longum*, *Lactobacillus helveticus*, *Lactococcus lactis*, and *Streptococcus thermophilus*). After one month, all groups experienced significant reductions in weight, BMI, and waist circumference. Interestingly, only the prebiotic and probiotic groups showed substantial decreases in fat mass, though differences between the groups were not statistically significant [117]. A recent study on 58 obese Egyptian women showed that probiotic intake combined with a hypocaloric diet with adequate fiber improves body composition, aids in weight loss, and normalizes serum leptin and AST levels [118].

Prebiotics are non-digestible ingredients in food that specifically feed beneficial gut bacteria. Examples include inulin, fructooligosaccharides (FOS), and galactooligosaccharides (GOS). These compounds help improve gut microbiota composition and strengthen the gut barrier [119,120,121]. By serving as fuel for beneficial microbes, prebiotics also encourage the production of short-chain fatty acids (SCFAs) like butyrate and acetate, which are essential for gut health and immune support [122,123,124]. A review of studies on prebiotic supplementation in patients with non-alcoholic fatty liver disease and diabetes mellitus showed inconsistent effects on body weight and BMI. While three studies indicated weight loss, two reported reductions in BMI. These varying outcomes highlight that the effectiveness of prebiotics may depend on factors like dosage, treatment duration, and individual patient differences [125].

Synbiotics are combinations of probiotics and prebiotics designed to amplify their combined effects. They have been shown to benefit conditions like irritable bowel syndrome (IBS) and inflammatory bowel disease (IBD) while also improving metabolic health [126,127,128,129]. Synbiotics can strengthen the gut barrier, reduce inflammation, and modulate immune responses, making them a promising area for further clinical research [130,131,132]. Evidence suggests that synbiotics may also potentiate the secretion of sIgA, thereby strengthening the gut’s immune defenses and supporting homeostasis [87]. Studies have highlighted the potential of synbiotics in addressing obesity-related parameters. A randomized, double-blind, placebo-controlled trial by Sergeev et al. (2020) evaluated the effects of a synbiotic supplement containing *Lactobacillus gasseri* and inulin on gut microbiota, body composition, and weight loss in overweight and obese adults. The study demonstrated significant improvements in gut microbial diversity, reductions in body weight, and favorable changes in body fat composition compared to the placebo group. These results highlight the synergistic potential of probiotics and prebiotics in managing obesity and improving metabolic health [133]. Similarly, an RCT conducted on overweight adults showed that *Lactobacillus gasseri* and synbiotic (probiotic with GOS) interventions significantly altered gut microbiota composition and improved metabolic parameters, including reduced inflammation and enhanced weight management [134]. Another study by Crovesy et al. (2021) demonstrated that a synbiotic containing *Bifidobacterium lactis* and FOS significantly modulates gut microbiota, reduces serum inflammatory markers, and improves metabolic health in obese women [135].

Research evidence supports the role of probiotics, prebiotics, and synbiotics in mitigating microbial dysbiosis, enhancing immune function, and reducing gastrointestinal inflammation [136]. Clinical applications are growing, but limitations, such as small sample sizes and short study durations, challenge the evaluation of long-term effects and safety [137]. Comparative research is needed to determine the most effective interventions and to optimize dosage schemes. Furthermore, more comprehensive studies are required to explore the role of combined interventions and to understand their specific effects on sIgA and intestinal health.

An imbalance in the gut microbiota, known as microbial dysbiosis, is influenced by antibiotic use, dietary habits, and bacterial infections. Dysbiosis is associated with gastrointestinal conditions like IBS, celiac disease, and antibiotic-associated diarrhea (AAD) [119,120,121,138,139].

Probiotics have proven effective in treating AAD by mitigating the effects of *Clostridioides difficile*, a major contributor to this condition [120,121]. Furthermore, probiotics have shown benefits in managing IBD, such as ulcerative colitis (UC), while being less effective for Crohn’s disease (CD) [124,126]. These findings suggest that maintaining gut microbial balance is crucial in managing these conditions.

Obesity is increasingly linked to alterations in gut microbiota, which affect metabolic processes, inflammation, and glucose metabolism [127,128,129]. Gut bacteria, particularly those from the phylum Bacteroidetes, play a role in glucose intolerance, a precursor to metabolic disorders such as diabetes and cardiovascular disease [128,129]. Reduced bacterial diversity and specific microbial profiles are associated with higher risks of insulin resistance, metabolic syndrome, and obesity [140].

Clinical interventions using probiotics have demonstrated promising results in managing obesity-related parameters. For instance, supplementation with *Lactobacillus plantarum* has been shown to reduce cholesterol and triglyceride levels. At the same time, synbiotic interventions combining probiotics with prebiotics have improved glucose regulation and reduced body fat mass [133,135,141]. Bariatric surgery, known to modify the gut microbiota, further highlights the relationship between microbial composition and weight regulation [142,143,144].

### 6.2. Micronutrients and Gut Health

Micronutrients, including vitamins and minerals, are essential for sustaining metabolism, bolstering immunity, and supporting cellular functions. The gut microbiota plays a pivotal role in the synthesis and regulation of key vitamins, particularly B vitamins (e.g., B1, B2, B6, B9, and B12) and vitamin K [145,146,147,148]. For instance, microbial production of folate (B9) in the colon not only surpasses dietary intake but also forms a critical component of the host’s metabolic network [146,147]. This relationship exemplifies the symbiotic dynamics between microbes and their host. However, the competition for nutrients like vitamin B12 adds another layer of complexity, as certain gut bacteria can sequester this vital micronutrient, potentially influencing both microbial composition and host health [149,150,151]. Fat-soluble vitamins, such as A, D, E, and K, play a critical role in immune responses and gut barrier integrity. Vitamin D, for instance, indirectly influences gut microbiota by modulating host immune functions, while vitamin A contributes to colonocyte health and may reduce colon cancer risk [152,153,154].

Trace elements like calcium, iron, and dietary polyphenols play pivotal roles in maintaining gut health. Epidemiological data suggest that increased calcium intake is associated with a decreased risk of obesity, potentially due to its effects on lipid metabolism and gut barrier integrity [155]. However, excessive iron supplementation, particularly in individuals with IBD, can disturb the delicate balance of gut microbiota, leading to increased inflammation and microbial dysbiosis [156,157,158,159]. Polyphenols—bioactive compounds in green tea, red wine, and dark chocolate—offer significant benefits by selectively enhancing the growth of beneficial gut bacteria such as *Lactobacillus* and *Bifidobacterium* [160]. These compounds improve microbial diversity, inhibit harmful bacteria, and contribute to producing SCFAs, which support immune function and gut health [161,162,163,164,165,166,167].

Studies have shown that iron supplementation raises colorectal cancer and intestinal disease risk [156]. Iron supplementation is a common intervention for iron deficiency-induced anemia, but its effects on gut health depend heavily on the dosage, the mode of administration, and the individual’s underlying health. Excessive or poorly regulated iron supplementation has been associated with adverse outcomes, including increased risk of gut inflammation and dysbiosis. For example, oral iron supplementation in a colitis rat model has been shown to worsen intestinal inflammation [157]. Similarly, in infants, high doses of iron were linked to increased markers of inflammation and gut microbial imbalance, suggesting a dose-dependent effect on gut health [158]. In individuals with inflammatory bowel disease (IBD), oral iron supplementation has been found to worsen mucosal inflammation compared to intravenous iron, which bypasses the gut and mitigates these adverse effects [159]. These findings suggest that the form and dosage of iron supplementation play critical roles in determining its safety and efficacy.

### 6.3. Challenges and Future Directions

Despite the promising potential of dietary strategies, probiotics, and synbiotics, there are still challenges. Most studies in this field have small sample sizes and short durations, making it hard to draw definitive conclusions about long-term safety and effectiveness. Additionally, comparative research is lacking and is required to identify the most effective interventions and the best combinations and dosages for clinical use. Addressing these gaps is crucial for creating effective intervention strategies. The interplay between diet, nutrients, and gut microbes is vital in health and disease prevention. While current research highlights the benefits of probiotics, prebiotics, and specific nutrients for managing gut-related issues, more robust studies are needed to fully understand their mechanisms and optimize their use in clinical settings.

## 7. Conclusions and Future Perspectives

Malnutrition, encompassing undernutrition, overnutrition, and micronutrient deficiencies, remains a significant global challenge, particularly driven by poverty and food insecurity. Maternal malnutrition during pregnancy and breastfeeding is critical, as it affects both the mother’s and child’s microbiota, especially during the first 1000 days of life—a period essential for healthy microbiota maturation. Deficiencies in key micronutrients can hinder this development, impacting growth and immune function in adulthood [168].

Undernourished individuals, especially children, often exhibit less diverse gut microbiota dominated by pathogenic bacteria, exacerbating nutrient deficiencies and weakening the immune system [66]. This dysbiosis impairs nutrient absorption and increases susceptibility to gastrointestinal infections due to lower levels of beneficial bacteria like *Lactobacillus* and *Bifidobacterium* [169]. Severe protein and micronutrient deficiencies can compromise gut barrier integrity and reduce antimicrobial peptide production, leading to issues like diarrhea and chronic inflammation [170].

Conversely, obesity—another form of malnutrition—is associated with a disrupted gut microbiota characterized by reduced levels of beneficial bacteria such as *Akkermansia muciniphila*. This imbalance can lead to increased gut permeability (“leaky gut”), systemic inflammation, and enhanced calorie extraction from food, perpetuating weight gain and metabolic disorders [171]. Notably, high-fat diets in animal studies have shown elevated LPS levels that contribute to inflammation and metabolic dysfunction [172].

Secretory immunoglobulin A (sIgA) maintains gut health and microbial balance. Reduced sIgA levels correlate with lower microbial diversity, making individuals more prone to dysbiosis and related conditions like IBD and IBS [83]. Encouragingly, dietary interventions—such as consuming probiotic-rich foods like yogurt and kimchi—can boost sIgA production, fostering a healthier, more resilient microbiota [100].

In conclusion, addressing malnutrition requires a holistic understanding of gut health. Promoting microbiota diversity through proper nutrition and supporting sIgA levels can mitigate the adverse effects of both undernutrition and obesity, ultimately enhancing immune function and overall well-being [66]. Future studies might create customized diets or probiotic therapies to increase sIgA production and promote microbial diversity. This strategy might be especially helpful for vulnerable groups, such as children in malnourished areas. A possible approach to re-establishing a balanced microbiota is the development of microbiota-targeted treatments, such as next-generation probiotics or FMT [173].

Future studies should concentrate on maternal and infant nutrition because the first 1000 days of life significantly impact gut microbiota development. Optimizing sIgA levels and microbiota composition through proper dietary intake during pregnancy and breastfeeding may prevent malnutrition-related long-term health problems. Future research should examine dietary nutritional elements and nutrients promoting sIgA secretion. For instance, studying how prebiotics, SCFAs, and certain micronutrients like zinc and vitamin A contribute to forming sIgA may result in new dietary recommendations and supplements.

Preventative measures may be made possible by understanding how sIgA–microbiota interactions affect the onset of conditions like obesity, metabolic syndrome, and IBD. The risk of these disorders may be decreased by raising sIgA levels through diet or microbiota modification, especially in malnourished populations. A deeper understanding of the molecular mechanisms at work will be made possible by investigating the interactions between malnutrition, gut microbiota, and sIgA using multi-omics techniques (genomics, proteomics, and metabolomics). This might make it easier to find biomarkers for the early identification of dysbiosis linked to malnutrition and direct-focused therapies.

Future initiatives must aim to integrate microbiota research into international health strategies. By guaranteeing access to nutrient-rich diets, probiotics, and educational initiatives, the detrimental effects of malnutrition on sIgA production and gut health can be lessened in low-income areas. Addressing how malnutrition affects sIgA–microbiota interactions will require a multidisciplinary strategy that combines cutting-edge research with useful, community-based treatments. By utilizing early-life interventions, microbiota-targeted medicines, and tailored diets, we can promote better gut environments and increase overall immunological resilience in vulnerable groups.

## Figures and Tables

**Table 1:** Studies evaluating the effects of undernutrition on sIgA and gut microbiota.

Study	Population/Model	Key Findings	Reference
Reddy et al. (1976)	Children with protein–calorie malnutrition	Significant reduction in secretory IgA in duodenal fluid, saliva, nasal secretions, and tears.	[72]
Subramanian et al. (2014)	Malnourished Bangladeshi children	Persistent gut microbiota immaturity associated with malnutrition.	[52]
Kane et al. (2015)	Pediatric malnutrition	Malnutrition impairs gut immune responses and reduces microbial diversity, weakening the gut barrier.	[59]
Monira et al. (2011)	Bangladeshi children	Malnourished children show altered gut microbiota composition, with reduced beneficial bacteria like *Lactobacillus*.	[67]
Rytter et al. (2014)	Children with malnutrition	Impaired immune responses and increased susceptibility to infections due to reduced sIgA levels.	[73]

**Table 2:** Studies investigating the impact of overnutrition and obesity on sIgA and gut microbiota.

Study	Population/Model	Key Findings	Reference
Ley et al. (2006)	Obese humans	Obesity is associated with decreased microbial diversity and increased capacity for energy harvest.	[42]
Turnbaugh et al. (2006)	Mice with diet-induced obesity	High-fat diets lead to gut dysbiosis.	[75]
Everard et al. (2013)	Mice with diet-induced obesity	*Akkermansia muciniphila* abundance is inversely correlated with obesity and inflammation.	[79]
Ridaura et al. (2013)	Twins discordant for obesity	Gut microbiota from obese twins induced greater adiposity in germ-free mice than in lean twins.	[81]

**Table 3:** Comparative effects of undernutrition and overnutrition on sIgA–microbiota interactions.

Aspect	Undernutrition	Overnutrition/Obesity
sIgA Levels	Decreased due to protein–calorie malnutrition and reduced immune function.	Decreased due to chronic low-grade inflammation and microbiota dysbiosis.
Microbial Diversity	Lower diversity and loss of beneficial species like *Lactobacillus* and *Bifidobacterium*.	Lower diversity and dominance of pro-inflammatory species like *Firmicutes*.
Gut Barrier Integrity	Compromised due to reduced sIgA and immune dysfunction.	Compromised by inflammation-induced permeability and reduced mucosal defenses.
Key Mechanisms	Nutrient deficiency impairs IgA production pathways (e.g., TGF-β and NF-κB signaling).	High-fat diets induce dysbiosis, metabolic endotoxemia, and inflammatory cytokine release.
Intervention Effects	Protein supplementation restores microbial balance and increases sIgA.	Dietary modifications (e.g., prebiotics and increased fiber) improve microbial diversity and gut health.

## Abbreviations

AAD	antibiotic-associated diarrhea
BMI	body mass index
CD	Crohn’s disease
EED	environmental enteric dysfunction
FMT	fecal microbiota transplantation
HOMA-IR	homeostatic model assessment of insulin resistance
IBD	inflammatory bowel diseases
IBS	irritable bowel syndrome
LPK	Lactobacillus plantarum K50
LPS	lipopolysaccharides
RYGB	Roux-en-Y gastric bypass
SCFAs	short-chain fatty acids
sIgA	secretory immunoglubulin A
UC	ulcerative colitis
WHO	World Health Organization
